# Impact of Alcohol Consumption on Cardiovascular Events in Patients Undergoing Percutaneous Coronary Intervention

**DOI:** 10.3390/jcm13216542

**Published:** 2024-10-31

**Authors:** Junpil Yun, Kyungdo Han, You-Jeong Ki, Doyeon Hwang, Jeehoon Kang, Han-Mo Yang, Kyung Woo Park, Hyun-Jae Kang, Bon-Kwon Koo, Hyo-Soo Kim, Jung-Kyu Han

**Affiliations:** 1 Cardiovascular Center, Seoul National University Hospital, Seoul 03080, Republic of Korea; junpilyun@gmail.com (J.Y.); cardiol.intv@gmail.com (D.H.); medikang@gmail.com (J.K.); hanname@hanmail.net (H.-M.Y.); kwparkmd@snu.ac.kr (K.W.P.); nowkang@snu.ac.kr (H.-J.K.); bkkoo@snu.ac.kr (B.-K.K.); hyosoo@snu.ac.kr (H.-S.K.); 2 Department of Statistics and Actuarial Science, Soongsil University, Seoul 06978, Republic of Korea; hkd917@naver.com; 3 Uijeongbu Eulji Medical Center, Uijeongbu-si 11749, Gyeonggi-do, Republic of Korea; drkiyou@gmail.com; 4 Department of Internal Medicine, College of Medicine, Seoul National University, 101 Daehak-ro, Jongno-gu, Seoul 03080, Republic of Korea

**Keywords:** PCI, alcohol consumption, cardiovascular risk

## Abstract

**Background/Objectives:** The impact of alcohol consumption and its restriction on clinical outcomes in patients undergoing percutaneous coronary intervention (PCI) remains elusive. We aimed to investigate the clinical outcomes in drinkers undergoing PCI. **Methods:** We included 77,409 patients who underwent PCI and a health check-up within one year of the PCI using a nationwide prospective database from the Korean National Health Insurance System. Primary outcomes were major adverse cardiovascular and cerebrovascular events (MACCE), a composite of all-cause mortality, myocardial infarction, coronary revascularization, and stroke. Patients were classified as non-drinkers, within-the-guideline (≤1 standard drink in women and ≤2 in men), and above-the-guideline drinkers based on drinking status at the first health check-up after PCI. **Results:** During a 4.0-year follow-up duration, MACCE incidence was 19.7% (*n* = 15,214) (4689 [6.1%] deaths, 1916 [2.5%] MI, 2033 [2.6%] strokes, and 10,086 [13.0%] revascularizations). Both within-the-guideline- (aHR [95%CI], 0.843 [0.773–0.919]) and above-the-guideline drinkers (0.829 [0.784–0.876]) had a lower MACCE risk than the non-drinkers. A characteristic J-curve relationship was observed between the frequency or body weight-adjusted alcohol consumption and MACCE risk, with the lowest risk in the once-per-week and a mild amount per body weight (≤0.33 g/kg/week) group. Drinking habits after PCI were associated with a lower risk of adverse cardiovascular outcomes; those who continued to drink before and after PCI had the lowest risk. **Conclusions:** Alcohol consumption was associated with a lower risk of adverse outcomes in patients undergoing PCI. Further studies with longer-term follow-up are warranted.

## 1. Introduction

Current guidelines recommend limiting alcohol intake to no more than one drink a day for women and two drinks a day for men to reduce cardiovascular risk in patients with stable coronary artery disease, non-ST-segment elevation acute coronary syndrome, or ST-segment elevation myocardial infarction (MI) [1,2,3]. Many observational studies reported a so-called J-curve relationship between alcohol drinking and cardiovascular events, with a lower risk for light-to-moderate drinkers compared with non-drinkers and a greater risk for heavy drinkers [2,4]. However, recent Mendelian randomization studies did not find the protective effects of moderate alcohol intake for cardiovascular health, compared with no or lower alcohol consumption [5,6]. This finding challenged the general concept that moderate alcohol drinking is associated with a lower risk of cardiovascular disease [7].

The most recent cardiovascular prevention guidelines limit alcohol consumption to 100 g per week, which is roughly equivalent to one to two drinks per day [7]. These recommendations still lack strong supporting evidence, and randomized controlled trials on this subject cannot be performed owing to ethical concerns. Moreover, the impact of alcohol consumption and its restriction on clinical outcomes in patients undergoing percutaneous coronary intervention (PCI) has not yet been elucidated. Herein, we conducted a large-scale population study based on the Korean National Health Insurance System (NHIS) to provide evidence on this subject.

## 2. Methods

### 2.1. Data Source and Study Participants

**Figure 1:**
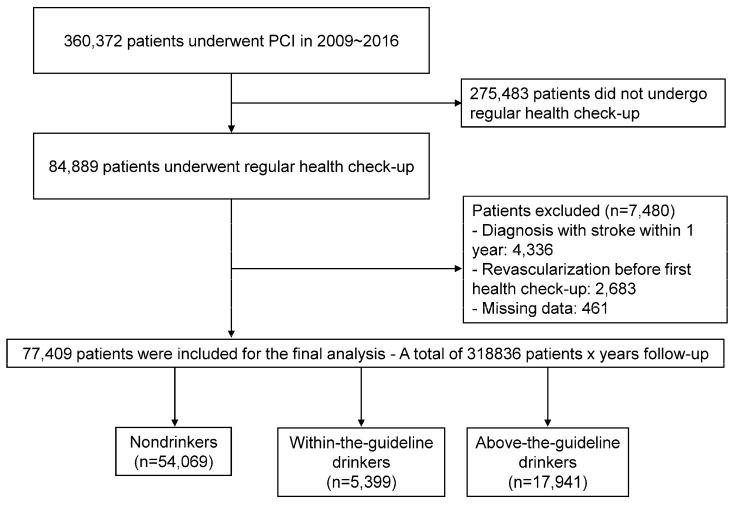
Study flow chart. We included 77,409 patients who underwent PCI and a health checkup within one year of the index PCI. Patients were classified as non-drinkers, within-the-guideline (≤1 standard drink in women and ≤2 in men), and above-the-guideline drinkers based on drinking status at the first health check-up after PCI. PCI, percutaneous coronary intervention.

We analyzed data from the Korean NHIS database. This database includes claims, health check-ups, and death information for people in the Republic of Korea. It includes baseline demographic information, diagnostic codes for diseases, data on in-patient and out-patient service usage, medication prescriptions, and mortality records. The NHIS serves as the sole insurer in the Republic of Korea, covering 97% of the South Korean population. All Koreans enrolled in the NHIS are recommended to undergo regular health check-ups at least every alternate year. We enrolled 84,889 patients who underwent a health check-up within 1 year after the index PCI, among 360,372 patients who underwent PCI between 1 January 2009 and 31 December 2016. To avoid confounding by pre-existing conditions, we excluded those with a history of stroke (International Classification of Diseases [ICD]-10 I63, 64) within one year after the index PCI (*n* = 4336) and those who underwent redo PCI before the first regular health check-up (*n* = 2683). After excluding those with missing data on alcohol consumption (*n* = 461), the final study population consisted of 77,409 individuals (Figure 1). The follow-up duration was from the date of the health check-up to the date of the occurrence of any clinical event or 31 December 2017, whichever occurred earlier. This study was conducted in compliance with the Declaration of Helsinki and received approval from the Institutional Review Board of the Seoul National University Hospital. Informed consent was waived for this study, as it relied on the anonymized database created by the NHIS.

### 2.2. Measurements and Definitions

Information on current alcohol consumption was obtained using a self-reported questionnaire. The questionnaire collected information on two parameters: (1) the number of times of alcohol consumption per week (frequency) and (2) the number of standard drinks (cups) consumed per drinking session. A standard drink was defined as a specialized cup for each type of alcohol, such as beer, whiskey, or a Korean distilled beverage (soju). Each cup of alcohol contained a similar amount of alcohol (8 g) [8]. The total alcohol consumption per week was calculated by multiplying the above two parameters. Above-the-guideline drinking was defined as consuming more than one standard drink (8 g of alcohol) per day for women and more than two drinks (16 g of alcohol) per day for men. Those who did not exceed these limits were categorized as within-the-guideline drinkers. 

To adjust the amount of alcohol consumption for body weight, the study population was categorized into six groups according to quintiles of alcohol consumption per body weight as follows: 0, non-drinker; Q1, ≤0.33 g/kg/week; Q2, 0.33–0.63 g/kg/week; Q3, 0.63–1.09 g/kg/week; Q4, 1.09–2.11 g/kg/week; and Q5, >2.11 g/kg/week. To determine the effect of alcohol cessation, we analyzed alcohol consumption trends of patients who underwent two serial regular health check-ups and questionnaires regarding alcohol consumption before and after the index PCI (*n* = 50,792). Persistent non-drinkers were used as reference. Smoking status was classified as a non-smoker, ex-smoker, or current smoker. Body mass index (BMI) was calculated by dividing body weight into kilograms by height in square meters. BMI classifications were as follows: low weight (BMI ˂ 18.5 kg/m^2^), normal weight (18.5–23.0 kg/m^2^), overweight (23.0–25.0 kg/m^2^), mildly obese (25.0–30.0 kg/m^2^), and morbidly obese (≥30.0 kg/m^2^). A person who exercised regularly was defined as a person who exercised ≥five times a week with moderate intensity or ≥three times a week with high intensity. 

### 2.3. Outcomes

The primary outcomes were major adverse cardiovascular and cerebrovascular events (MACCE), a composite of all-cause mortality, MI, coronary revascularization, and stroke. The secondary outcomes included the individual components of MACCE. MI was defined as the recording of ICD-10 codes I21 or I22 with a procedural code for coronary revascularization. Revascularization was defined using the procedural code for PCI or coronary artery bypass graft surgery. Stroke was defined as the recording of ICD-10 codes I63 or I64 during hospitalization, along with claims for brain imaging studies such as brain computed tomography and magnetic resonance imaging. MACCE risk was stratified in accordance with alcohol consumption (non-drinkers, within-the-guideline-, or above-the-guideline drinkers). The impact of both the frequency and amount of alcohol consumption per week was evaluated.

### 2.4. Statistical Analysis

All numerical data were presented as mean ± standard deviation for continuous variables and as percentages for categorical variables. The incidence rate for each outcome was determined as the number of events divided by the follow-up duration (per 1000 person-years). If combined end-points occurred in a patient, the first event was recorded. The Kaplan–Meier method was used to estimate the rate of time-dependent events, and clinical outcomes were compared using the log-rank test. To reduce the impact of potential confounding factors, we applied an inverse probability-weighted (IPW) Cox regression with a robust variance estimator. The probability was estimated using a multinomial logistic regression model with age, sex, hypertension, diabetes mellitus, dyslipidemia, body mass index, social income, regular exercise, and smoking status. The results were presented as adjusted hazard ratios (aHRs) with 95% confidence intervals (CIs) for end-points. In the subgroup analysis, aHR and 95% CI of the within-the-guideline or above-the-guideline drinker groups were compared with those of the non-drinker group. A two-sided *p*-value of less than 0.05 was considered statistically significant for all analyses. Statistical analyses were conducted using Statistical Analysis Software version 9.4 (SAS Institute, Cary, NC, USA).

## 3. Results

### 3.1. Baseline Characteristics of the Study Population

In total, 77,409 patients were included in the analysis. According to the self-reported drinking status at the first health check-up following the index PCI, there were 54,069 (69.8%) non-drinkers, 5399 (7.0%) within-the-guideline drinkers, and 17,941 (23.2%) above-the-guideline drinkers. The median age of the study cohort was 63 (Q1–Q3: 55–70) years, and 58,457 patients (75.5%) were men. Table 1 shows the baseline characteristics of each drinking group. Non-drinkers were older, more likely to be female, shorter, and less obese. This group had a higher prevalence of hypertension and diabetes mellitus, less regular physical activity, and a lower income. Within-the-guideline drinkers had a lower prevalence of hypertension and diabetes mellitus, exercised more regularly, and earned a higher income. Above-the-guideline drinkers were younger, more likely to be male, taller, and more obese. They also exhibited higher serum glucose, total cholesterol, and triglyceride levels. The proportion of current smokers was higher among patients who consumed more alcohol.

### 3.2. Alcohol Consumption Status and Clinical Outcomes

**Figure 2:**
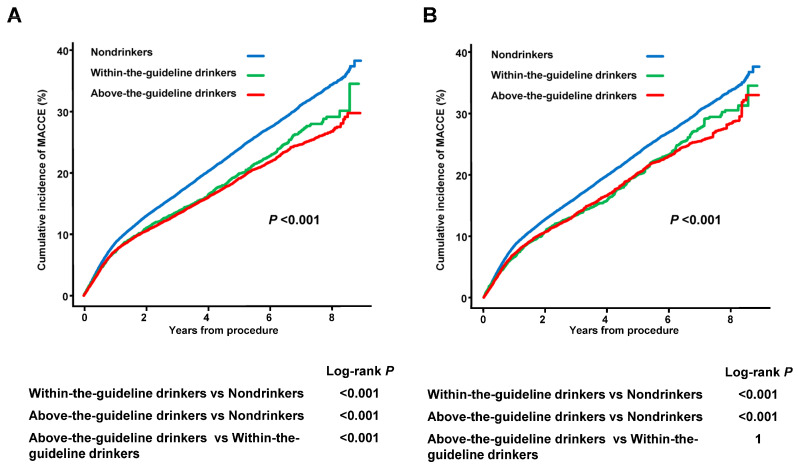
The Kaplan–Meier Curves for MACCE According to Drinking Status in Crude (**A**) and IPW-matched Populations (**B**). The Kaplan–Meier curves demonstrated the highest MACCE incidence in non-drinkers and the lowest in above-the-guideline drinkers in both the crude and IPW populations (log-rank *p* < 0.001). *n* = 77,409. A log-rank test was utilized for statistical analyses. IPW, inverse probability-weighted; MACCE, major adverse cardiovascular and cerebrovascular events.

During the 4.0 years of median follow-up, MACCE incidence was 19.7% (*n* = 15,214), including 4689 (6.1%) deaths, 1916 (2.5%) cases of MI, 2033 (2.6%) strokes, and 10,086 (13.0%) revascularizations (Table 2). Intriguingly, a negative relationship was observed between the drinking status and MACCE incidence. After IPW adjustment for demographic and lifestyle factors, within-the-guideline drinkers had a 15.7% lower MACCE risk (aHR 0.843, 95% CI 0.773–0.919) and above-the-guideline drinkers had a 17.1% lower MACCE risk (aHR 0.829, 95% CI 0.784–0.876) than the non-drinkers. The Kaplan–Meier curves demonstrated the highest MACCE incidence in non-drinkers and the lowest in above-the-guideline drinkers in both the crude and IPW populations (log-rank *p* < 0.001) (Figure 2). This interesting drinking status-dependent finding in MACCE was mainly driven by the significantly lower MI risk in within-the-guideline drinkers and especially in above-the-guideline drinkers. Notably, drinking was also associated with a significantly lower risk of death or stroke compared with non-drinking, and within-the-guideline drinkers showed the lowest risk.

### 3.3. Frequency and Body Weight-Adjusted Amount of Drinking and Clinical Outcomes

**Figure 3:**
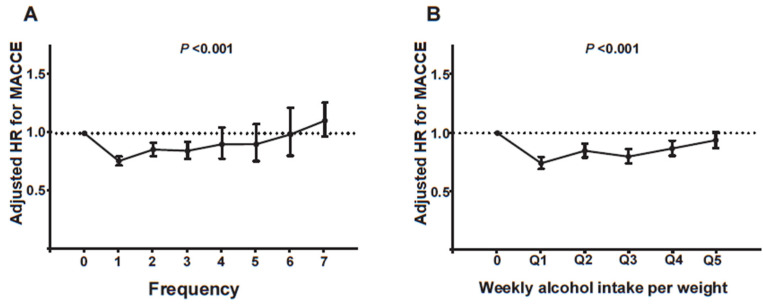
MACCE risk according to frequency (times per week) (**A**) and bodyweight-adjusted amount of drinking (**B**). Drinking frequency, defined by the number of drinks consumed per week, showed a J-curve relationship with the IPW-aHR of MACCE. The MACCE risk was the lowest in patients who drank alcohol once a week and gradually increased with alcohol intake frequency. (**A**) When weekly alcohol intake per body weight was divided into quintiles, a J-curve relationship was also found between body weight-adjusted drinking amount and clinical outcomes. (**B**) Q1, ≤0.33g/kg/week; Q2, 0.33g/kg/week ˂ to ≤0.63g/kg/week; Q3, 0.63g/kg/week ˂ to ≤1.09g/kg/week; Q4, 1.09g/kg/week ˂ to ≤2.11g/kg/week; Q5, >2.11 g/kg/week. *n* = 77,409. The Multivariable Cox regression model was utilized for statistical analyses. HR, hazard ratio; IPW, inverse probability-weighted; MACCE, major adverse cardiovascular and cerebrovascular events.

A more detailed analysis of the relationship between drinking frequency or body weight-adjusted drinking amount and clinical outcomes was performed. Interestingly, drinking frequency, defined by the number of drinks consumed per week, showed a characteristic J-curve relationship with the IPW-aHR of MACCE (Figure 3A). The MACCE risk was the lowest in patients who drank alcohol once a week and gradually increased with alcohol intake frequency. Notably, patients who drank daily exhibited a non-significant tendency to have a higher MACCE risk than non-drinkers (daily drinkers vs. non-drinkers; aHR, 1.108; 95% CI, 0.974–1.260).

Although above-the-guideline drinkers (>one standard drink daily in women and >two drinks in men) were associated with a lower MACCE risk (Table 2), the analysis using bodyweight-adjusted drinking amounts revealed a more intriguing picture. When weekly alcohol intake per body weight was divided into quintiles, each quintile showed a lower MACCE risk (Figure 3B). Notably, a J-curve relationship was also found between bodyweight-adjusted drinking amount and clinical outcomes. The first quintile (≤0.33 g/kg of body weight alcohol per week) was associated with the lowest MACCE risk, and the risk gradually increased with increased body weight-adjusted alcohol intake. However, the last quintile (>2.11 g/kg/week) was still associated with a lower MACCE risk than the non-drinkers.

### 3.4. Impact of Drinking Status After Revascularization on Clinical Outcomes

Interestingly, drinking habits after PCI were associated with a lower MACCE risk than non-drinking status after PCI (Table 3). Notably, alcohol abstinence after PCI was not associated with a lower MACCE risk. In contrast, persistent drinking before and after PCI was associated with the lowest MACCE risk (persistent drinkers vs. persistent non-drinkers; aHR, 0.777; 95% CI, 0.735–0.821).

### 3.5. Subgroup Analysis of MACCE

**Figure 4:**
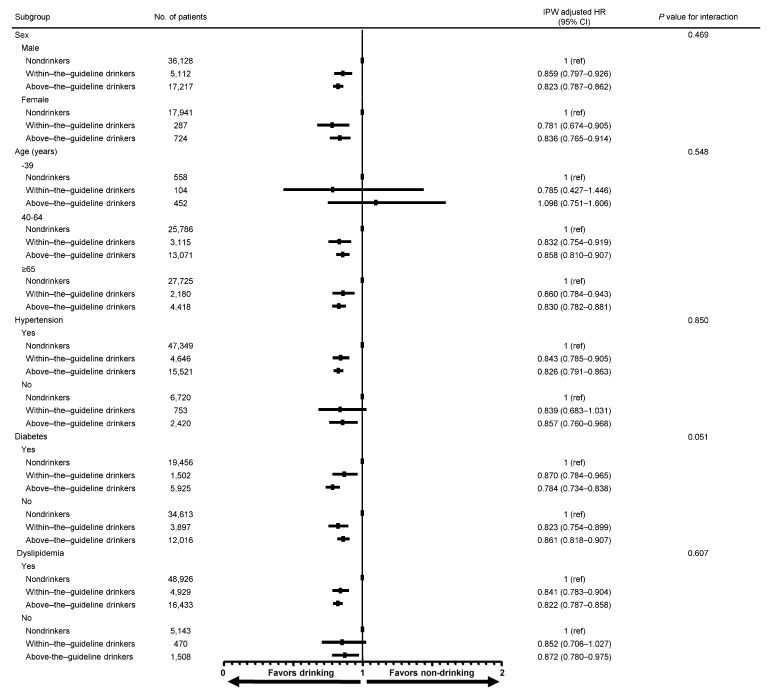
Subgroup analysis for MACCE. Lower drinking habit-associated MACCE risk was consistently observed across various subgroups, including sex, age, hypertension, diabetes mellitus, and dyslipidemia in the IPW-matched population. *n* = 77,409. A weighted Cox regression model was utilized to produce the *p*-values. MACCE, major adverse cardiovascular and cerebrovascular events.

Lower drinking habit-associated MACCE risk was consistently observed across various subgroups, including sex, age, hypertension, diabetes mellitus, and dyslipidemia in the IPW-matched population (Figure 4). The impact of alcohol consumption on better clinical outcomes in patients aged <40 years was not as apparent as that in patients aged ≥40 years. However, the sample size for this age group was too small to draw sound conclusions.

## 4. Discussion

Overall, the main findings of this large-scale population study, including 77,409 patients from the Korean NHIS database, are as follows: (1) drinkers, including those who drink above the level recommended by the current guidelines, were associated with a lower risk of adverse clinical outcomes after PCI; (2) a J-curve relationship was observed between the frequency or body weight-adjusted amount of alcohol intake and risk of cardiovascular outcomes, with the lowest risk in mild drinkers; and (3) drinking habits after PCI were associated with a lower risk of adverse cardiovascular outcomes, with the lowest risk in persistent drinkers who continued to drink before and after PCI.

### 4.1. Theoretical Benefits of Mild-to-Moderate Alcohol Consumption for Better Cardiovascular Outcomes

Meta-analyses have shown that moderate alcohol consumption (30 g alcohol/day) is associated with favorable changes in various cardiovascular biomarkers, including increased levels of high-density lipoprotein (HDL) cholesterol, adiponectin, and lower levels of fibrinogen [9,10,11]. A direct association between alcohol consumption and plasma level of hemostatic factors toward a more thrombolytic profile was also reported [9,12]. In addition, low-to-moderate alcohol consumption was suggested to improve insulin sensitivity and associated with decreased incidence of type 2 diabetes [13,14,15]. Insulin secretion is enhanced by low-to-moderate intake of alcohol, and alcohol oxidation may reduce gluconeogenesis [13,15]. Additionally, non-alcoholic components, such as polyphenols and other bioactive compounds, may contribute to the cardioprotective effects. Polyphenols are well-known for their antioxidants and anti-inflammatory properties, which positively impact cardiovascular health [16,17]. Wine known to be rich in polyphenols, when consumed in moderation, has been suggested to reduce depression and inflammation, which can potentially affect cardiovascular risk [16,18].

### 4.2. J-Curve Relationship Between Alcohol Drinking and Cardiovascular Outcomes

A characteristic J-curve relationship with the lowest risk among mild-to-moderate drinkers [19,20,21] or an inverse relationship [22,23] between drinking and cardiovascular outcomes has been reported in various studies, including prospective cohort studies, population-based studies, systemic reviews, and meta-analyses. A recent large-scale population-based cohort study also reported J-shaped associations for cardiovascular disease and all-cause mortality, with moderate drinkers having a lower risk than non-drinkers and heavy drinkers exceeding guidelines [24]. Interestingly, an inverse relationship was noted for MI and stable angina, with heavy drinkers having the lowest risk [24]. This study excluded former drinkers and occasional drinkers from non-drinkers to avoid a confounding effect by the “sick quitter” hypothesis [25], which states that patients would abstain from drinking for health reasons but still found a J-curve or inverse relationship.

In contrast, recent Mendelian randomized studies showed that lower alcohol consumption is associated with a lower risk of coronary heart disease or stroke, disputing the J-curve relationship [5,6]. This type of Mendelian randomized study fundamentally relies on the following assumptions [26,27]. First, the alleles of interest explain most variation in alcohol consumption. Second, groups classified by the presence of the alleles of interest have the same outcome risk except for the alleles themselves. Third, all effects of the alleles of interest on outcomes are mediated through alcohol consumption. Any violation of these core assumptions yields biased results.

One of the strengths of our population-based prospective cohort study with 77,409 patients was that it was based on the NHIS, the single insurer in South Korea, covering 97% of the population. Regular health check-ups using survey questionnaires regarding alcohol consumption were systematically conducted by the NHIS. Our study demonstrated the existence of a J-curve relationship among drinkers undergoing PCI. Intriguingly, everyday drinkers tended to have a higher MACCE risk in the J-curve for frequency of drinking, while the top quintile still had a lower MACCE risk in the J-curve for the amount of drinking than the non-drinkers. This finding suggests that constant exposure to alcohol could affect health more significantly than the amount of alcohol consumed. This interesting hypothesis should be evaluated in future studies. In this regard, a study reporting an inverse association between alcohol and MI showed that the risk remained consistent across different drinking frequency categories, regardless of the amount of alcohol consumed per drinking day [28].

### 4.3. MI and Non-Cardiovascular Risk Associated with Alcohol Consumption

A recent meta-analysis reported that the threshold for predicting all-cause mortality in current drinkers was 100 g/week [29]. This is roughly equivalent to the amount of alcohol recommended by current guidelines: 16 g/day for men and 8 g/day for women. This finding is largely consistent with our findings. In our study, within-the-guideline drinkers undergoing PCI had the lowest death risk, although above-the-guideline drinkers still had a lower death risk than non-drinkers. Notably, the meta-analysis found a J-shaped association between alcohol consumption and a composite of cardiovascular disease outcomes [29]. A detailed analysis revealed positive and roughly linear associations with cardiovascular diseases, excluding MI, such as stroke and coronary artery disease, whereas there was an inverse and approximately log-linear association with MI [29]. Interestingly, many previous studies have consistently reported an inverse relationship between alcohol and MI [22,23,24,29]. The Mendelian randomized study disputing the J-curve association between alcohol consumption and stroke also showed an inverse relationship tendency for MI, although it was not statistically significant [6]. Our study also demonstrated the lowest MI risk for above-the-guideline drinkers, in contrast to the lowest stroke risk for within-the-guideline drinkers. The underlying reasons for the inverse relationship, particularly between alcohol consumption and MI, are not fully understood. Alcohol intake is known to increase HDL cholesterol and blood pressure [6,29]. Given that HDL cholesterol is more associated with coronary disease than with stroke and that blood pressure is more associated with stroke than with coronary disease, the differential impact of alcohol on MI and stroke could be mediated by alcohol-induced upregulation of HDL cholesterol and blood pressure [6,29].

The harmful effects of alcohol on non-cardiovascular diseases, such as liver cirrhosis, injuries, and cancer, have been well established [5,20]. Therefore, a J-curve or inverse relationship between alcohol and cardiovascular outcomes could be offset by non-cardiovascular outcomes. Considering that our study population was composed of patients undergoing PCI, the outcome of death in our study could be mainly affected by cardiovascular diseases during the 4-year follow-up. Increased risks of non-cardiovascular diseases could offset the protective effect of alcohol on death observed in our study in longer-term follow-up.

### 4.4. Impact of Alcohol Consumption in Cardiovascular Secondary Prevention Setting

Mild-to-moderate alcohol consumption has been linked to a lower all-cause mortality risk in patients following MI [30,31] or those with cardiovascular disease [32]. Alcohol consumption was associated with reduced restenosis following percutaneous transluminal coronary angioplasty or stent implantation [33]. To our knowledge, our study is the first study to evaluate the impact of alcohol consumption and its restriction on the clinical outcomes of patients undergoing PCI. Our study, based on serial regular health check-up data, showed a lower MACCE risk in drinkers after PCI than in non-drinkers. Our study also found that patients who started drinking after PCI had a lower MACCE risk than the persistent non-drinkers.

### 4.5. Study Limitations

Our study had some limitations. First, the status and amount of alcohol consumption were referenced using self-reported questionnaires. It is possible that sick patients underreported their alcohol intake. However, a previous study demonstrated the reproducibility and validity of alcohol consumption measured by a self-administered questionnaire [34]. Second, we did not have information about the preferred types of alcoholic beverages. Because non-alcohol components such as polyphenols in fermented beverages may contribute to the cardioprotective effects, the impact of alcohol on cardiovascular outcome could be different according to the type of alcohol. However, one report about alcohol consumption by Koreans demonstrated that Korean distilled spirit (soju), which did not include a non-alcohol component, accounts for 66.5% of total alcohol consumption in Korea [35]. Third, there were intrinsic limitations of a non-randomized prospective cohort study, such as differences in the distribution of risk factors and potential influences from unmeasured confounding factors. In particular, drinkers were often associated with better baseline characteristics, such as more physical activity, higher socioeconomic status, and greater fruit intake [30,36]. In addition, sick patients would quit drinking for health reasons. Although we adjusted for multiple variables, including regular exercise and social income, using weighted Cox proportional hazard regression models with IPW, potential biases due to unmeasured confounding factors could not be overcome. However, because of ethical concerns, randomized controlled trials for alcohol consumption would not be feasible. In this regard, large-scale population-based studies such as ours can provide important evidence about alcohol drinking habits after PCI. Fourth, because factors determining alcohol distribution, such as body size and fat percentage, and genetic variants related to alcohol metabolism [6] vary in different ethnic groups, our findings could not be generalized to all ethnic groups. At least, we performed analyses for daily alcohol intake after normalization by body weight to overcome body size-related biases.

## 5. Conclusions

Based on a nationwide population database, our study revealed that alcohol consumption was associated with a lower risk of adverse cardiovascular events in patients after PCI. There was a J-curve relationship between alcohol consumption and clinical outcomes, with the lowest risk in mild drinkers. Mild drinking habits would be allowed for patients undergoing PCI, but further studies with longer-term follow-up are warranted.

## Figures and Tables

**Table 1:** Baseline characteristics.

	Total (*n* = 77,409)	Non-Drinker (*n* = 54,069)	Within-the-Guideline Drinker (*n* = 5399)	Above-the-Guideline Drinker (*n* = 17,941)	*p*-Value
Age, years					<0.001
20–39	1114 (1.4)	558 (1.0)	104 (1.9)	452 (2.5)	
40–64	41,972 (54.2)	25,786 (47.7)	3115 (57.7)	13,071 (72.9)	
65+	34,323 (44.4)	27,725 (51.3)	2180 (40.4)	4418 (24.6)	
Male	58,457 (75.5)	36,128 (66.8)	5112 (94.7)	17,217 (96.0)	<0.001
Hypertension	67,516 (87.2)	47,349 (87.6)	4646 (86.1)	15,521 (86.5)	<0.001
Diabetes mellitus	26,883 (34.7)	19,456 (36.0)	1502 (27.8)	5925 (33.0)	<0.001
Dyslipidemia	70,288 (90.8)	48,926 (90.5)	4929 (91.3)	16,433 (91.6)	<0.001
Smoking status					<0.001
Non-smoker	35,294 (45.6)	30,141 (55.7)	1660 (30.8)	3493 (19.5)	
Ex-smoker	29,808 (38.5)	17,512 (32.4)	2950 (54.6)	9346 (52.1)	
Current smoker	12,307 (15.9)	6416 (11.9)	789 (14.6)	5102 (28.4)	
Regular exercise	18,119 (23.4)	11,734 (21.7)	1585 (29.4)	4800 (26.8)	<0.001
Low income (lower quintile)	14,615 (18.9)	10,681 (19.8)	891 (16.5)	3043 (17.0)	<0.001
BMI, kg/m^2^					<0.001
Underweight (˂18.5)	1045 (1.4)	876 (1.6)	59 (1.1)	110 (0.6)	
Normal weight (18.5–23)	19,742 (25.5)	14,542 (26.9)	1396 (25.9)	3804 (21.2)	
Overweight (23–25)	21,399 (27.6)	14,863 (27.5)	1634 (30.2)	4902 (27.3)	
Obese (25–30)	31,524 (40.7)	21,300 (39.4)	2133 (39.5)	8091 (45.1)	
Morbidly obese (≥30)	3699 (4.8)	2488 (4.6)	177 (3.3)	1034 (5.8)	
Height, cm	163.4 ± 8.8	161.8 ± 9.1	166.6 ± 6.7	167.5 ± 6.5	<0.001
Weight, kg	66.4 ± 11.1	64.8 ± 11.1	68.4 ± 9.7	70.8 ± 10.5	<0.001
Waist circumference, cm	85.5 ± 8.0	85.0 ± 8.2	85.7 ± 7.4	87.0 ± 7.7	<0.001
*Laboratory findings*					
Hemoglobin, g/dL	13.8 ± 1.6	13.6 ± 1.6	14.3 ± 1.4	14.4 ± 1.3	<0.001
Fasting glucose, mg/dL	111.2 ± 35.2	111.1 ± 35.7	108.4 ± 33.8	112.4 ± 34.0	<0.001
Total cholesterol, mg/dL	150.1 ± 39.5	149.6 ± 37.8	148.5 ± 52.1	152.0 ± 40.2	<0.001
Triglyceride, mg/dL, median (IQR)	117.9 (117.5, 118.3)	114.4 (114.0, 114.9)	111.1 (109.6, 112.6)	129.0 (128.0, 130.1)	<0.001
*Blood pressure*					
Systolic blood pressure, mmHg	126.0 ± 15.7	126.1 ± 16.0	124.8 ± 15.1	126.1 ± 15.1	<0.001
Diastolic blood pressure, mmHg	76.3 ± 10.0	76.0 ± 10.1	76.1 ± 9.7	77.6 ± 9.9	<0.001

Figures are numbers (percentage) of patients for categorical variables or mean ± standard deviation for continuous variables unless stated otherwise. BMI = body mass index; IQR = interquartile range.

**Table 2:** Hazard ratios and 95% confidence intervals of MACCE, all-cause mortality, myocardial infarction, revascularization, and stroke by drinking status.

	Events (*n*)	Follow-Up Duration (Person-Years)	Incidence Rate (per 1000 Person-Years)	IPW Matched * Adjusted HR (95% CI)
MACCE				
Non-drinker	11,336	197,503	57.397	1 (ref)
Within-the-guideline drinker	929	19,806	46.905	0.843 (0.773 to 0.919)
Above-the-guideline drinker	2949	66,117	44.603	0.829 (0.784 to 0.876)
Death				
Non-drinker	3774	223,206	16.908	1 (ref)
Within-the-guideline drinker	243	22,048	11.022	0.734 (0.620 to 0.868)
Above-the-guideline drinker	672	73,582	9.133	0.750 (0.665 to 0.845)
Myocardial infarction				
Non drinker	1386	219,687	6.309	1 (ref)
Within-the-guideline drinker	143	21,711	6.587	0.968 (0.774 to 1.210)
Above-the-guideline drinker	387	72,532	5.336	0.744 (0.638 to 0.868)
Stroke				
Non-drinker	1601	219,538	7.293	1 (ref)
Within-the-guideline drinker	97	21,847	4.440	0.672 (0.522 to 0.866)
Above-the-guideline drinker	335	72,824	4.600	0.800 (0.677 to 0.946)
Revascularization				
Non-drinker	7237	200,558	36.084	1 (ref)
Within-the-guideline drinker	678	19,990	33.918	0.929 (0.839 to 1.028)
Above-the-guideline drinker	2171	66,721	32.539	0.867 (0.813 to 0.926)

CI = confidence interval; HR = hazard ratio; MACCE = Major adverse cardiovascular and cerebrovascular events; ref = reference * IPW model: Weights are calculated by age, sex, hypertension, diabetes mellitus, dyslipidemia, body mass index, social income, regular exercise, and smoking status.

**Table 3:** Incidence of MACCE according to drinking habit change after PCI.

Alcohol Status Pre PCI/Post PCI	*n*	Events (*n*)	Follow-Up Duration (Person-Years)	Incidence Rate (per 1000 Person-Years)	IPW * Adjusted HR (95% CI)
Non/Non	26,453	4761	85,227	55.863	1 (ref)
Non/Drinking	2467	384	8569	44.815	0.849 (0.775 to 0.930)
Drinking/Non	8291	1487	27,276	54.518	0.952 (0.900 to 1.008)
Drinking/Drinking	13,581	1906	44,363	42.964	0.777 (0.735 to 0.821)

CI = confidence interval; HR = hazard ratio; MACCE = Major adverse cardiovascular and cerebrovascular events; PCI = percutaneous coronary intervention. * IPW model: Weights are calculated by age, sex, hypertension, diabetes mellitus, dyslipidemia, body mass index, social income, regular exercise, and smoking status.

## Data Availability

The original contributions presented in the study are included in the article.

## Abbreviations

BMI	body mass index
CI	confidence interval
HDL	high-density lipoprotein
HR	hazard ratio
ICD	International Classification of Diseases
IPW	inverse probability-weighted
MACCE	major adverse cardiovascular and cerebrovascular events
MI	myocardial infarction
gNHIS	National Health Insurance System
PCI	percutaneous coronary intervention
